# In This Issue

**Published:** 2002

## COMORBIDITY OF ALCOHOLISM AND PSYCHIATRIC DISORDERS: AN OVERVIEW

Patients with co-occurring alcohol abuse or dependence and other psychiatric conditions present special challenges for treatment professionals. Although pharmacological and psychosocial treatments for alcohol use disorders and psychiatric disorders can be integrated to help these patients, relatively few clinical studies have tested the results of such treatments. Drs. Ismene L. Petrakis, Gerardo Gonzalez, Robert Rosenheck, and John H. Krystal report on the prevalence and treatment of co-occurring psychiatric and alcohol use disorders. The authors describe why medications and psychosocial treatments may be especially valuable for this patient population. (pp. 81–89)

## ALCOHOLISM AND PSYCHIATRIC DISORDERS: DIAGNOSTIC CHALLENGES

In evaluating an alcoholic patient who also suffers from psychiatric symptoms, the clinician must determine whether these are isolated symptoms related to the patient’s alcohol use, whether the symptoms indicate the presence of an alcohol-induced psychiatric disorder that will improve on its own once the client achieves abstinence, or whether the symptoms result from an independent psychiatric disorder that requires treatment. Drs. Ramesh Shivani, R. Jeffrey Goldsmith, and Robert M. Anthenelli propose a five-component approach to evaluating such patients. Those components include inquiring about alcohol use when evaluating psychiatric complaints, differentiating between alcohol-related symptoms and alcohol-induced mental disorders, distinguishing alcohol-induced and independent disorders, considering various patient characteristics, and remaining flexible with respect to diagnosis and followup. (pp. 90–98)

## CO-OCCURRING ALCOHOL USE DISORDER AND SCHIZOPHRENIA

Alcohol use disorder is extremely common among people with schizophrenia. Researchers have proposed several biological and psychosocial factors that may contribute to this co-occurrence. As Drs. Robert E. Drake and Kim T. Mueser explain, schizophrenia patients with alcohol use disorder are more likely to have social, legal, and medical problems, compared with other people with schizophrenia. Alcohol use disorder also complicates the course and treatment of schizophrenia. The authors describe how mental health treatment and substance abuse treatment approaches can be integrated to effectively treat this comorbid population. Integrated treatment emphasizes outreach, comprehensiveness, and a stage-wise approach to treatment and recovery. (pp. 99–102)

## BIPOLAR DISORDER AND ALCOHOLISM

Alcoholism and bipolar disorder commonly co-occur, and the presence of alcoholism may adversely affect the course and prognosis of bipolar disorder, making it more difficult to treat. Although it is unclear why these conditions co-occur, researchers have proposed that one disorder may contribute to the development of the other. Genetic risk factors common to both disorders also may play a role. Drs. Susan C. Sonne and Kathleen T. Brady describe the prevalence and diagnosis of comorbid bipolar disorder and alcoholism and review research on the appropriate treatment for these patients. Some studies have evaluated the effects of medications, as well as psychosocial interventions, in treating alcoholic bipolar patients, but further research is needed. (pp. 103–108)

## CHILDHOOD ANTISOCIAL BEHAVIOR AND ADOLESCENT ALCOHOL USE DISORDERS

Children with antisocial behavior and related mental disorders (i.e., conduct disorder and oppositional defiant disorder) are more likely to experience problems with alcohol during adolescence. Drs. Duncan B. Clark, Michael Vanyukov, and Jack Cornelius describe a model to explain the relationship between childhood antisocial behaviors and adolescent alcohol use disorders. This model posits common causes for both behaviors. For example, both antisocial behavior and alcohol use disorders may result from a person’s inability to control his or her behavior and/or from common genetic or environmental influences. This model has implications for the prevention, evaluation, and treatment of adolescent alcohol use disorders. Programs to diminish antisocial behavior during childhood may prevent the subsequent development of problems with alcohol. Adolescents with alcohol use disorders also should be assessed for the presence of antisocial behavior to select the most appropriate treatment approaches. (pp. 109–115)

## COMORBID PSYCHIATRIC FACTORS CONTRIBUTING TO ADOLESCENT ALCOHOL AND OTHER DRUG USE

Alcohol and other drug (AOD) use by adolescents is a major public health problem. To design effective measures for preventing this problem, it is crucial to understand the factors that influence adolescent AOD use. Drs. Deborah Deas and Suzanne Thomas explore these factors, including psychological, psychiatric, environmental, and peer and family influences. Psychological risk factors include personality characteristics (e.g., novelty seeking or aggressiveness), low self-esteem, and exposure to stressful and traumatic life events. Co-occurring psychiatric disorders include depression, anxiety disorders, conduct disorder, and attention deficit hyperactivity disorder. The relative impact that these and other risk factors have in the initiation and maintenance of adolescent AOD are likely to change over the course of adolescence. (pp. 116–121)

## THE CLINICALLY MEANINGFUL LINK BETWEEN ALCOHOL USE AND ATTENTION DEFICIT HYPERACTIVITY DISORDER

Attention deficit hyperactivity disorder (ADHD) is a childhood mental disorder characterized by inattention, impulsivity, and hyperactivity. As Drs. Bradley H. Smith, Brooke S.G. Molina, and William E. Pelham, Jr., report, some findings suggest that ADHD may play a role in the development of alcohol-related problems in adolescence and adulthood. For example, a clinically significant correlation exists between ADHD and developmentally inappropriate levels of alcohol use (i.e., early initiation of alcohol consumption during adolescence or alcohol abuse and dependence during young adulthood). The presence of ADHD in people with alcohol abuse or dependence has important implications for the treatment of those patients. Alcoholism treatment approaches for such patients must take into consideration their deficits in attention and impulse control. (pp. 122–129)

## SOCIAL ANXIETY DISORDER AND ALCOHOL USE

People suffering from social anxiety disorder have an excessive fear that they will do something embarrassing in social situations or show symptoms of anxiety (e.g., blushing or sweating) that will be humiliating. People with social anxiety disorder also have a higher incidence of alcohol use disorders (AUDs) compared with the rest of the population. Because people in general expect alcohol to alleviate stress and anxiety, it is possible that people with greater anxiety levels may consume more alcohol to help ease their fears. Drs. Sarah W. Book and Carrie L. Randall describe studies investigating people’s expectancies about alcohol’s ability to reduce social fears, studies that have determined how expectancies translate into actual alcohol consumption, and studies that demonstrated that alcohol reduces social fears in a laboratory setting. The authors also discuss treatment strategies that could be used for patients with co-occurring social anxiety disorder and AUDs, including various forms of pharmacotherapy and psychotherapy. (pp. 130–135)

## CONCURRENT ALCOHOL AND TOBACCO DEPENDENCE: MECHANISMS AND TREATMENT

People who drink alcohol also are likely to smoke; studies show that up to 90 percent of alcoholics are smokers. According to Dr. David J. Drobes, several factors may contribute to concurrent alcohol and tobacco use. For example, genetic factors that increase a person’s risk for problems with alcohol also may increase his or her risk for tobacco use. These genetic risk factors may be related to certain brain chemical systems, such as those involved in cross-tolerance to alcohol and tobacco use. Likewise, conditioning processes and psychosocial factors (e.g., personality characteristics, family influences, and comorbid psychological disorders) also may play a role. Although the most effective approach for the treatment of smoking alcoholics remains to be determined, several findings support concurrent treatment for both addictions. (pp. 136–142)

## PATHOLOGICAL GAMBLING AND ALCOHOL USE DISORDER

Studies show that alcohol use disorders and problem gambling often co-occur at a high rate, though little is known about how gender, race/ethnicity, age, and other psychiatric disorders influence this relationship. Regardless of the reasons for this comorbidity, the fact that these disorders frequently occur in tandem raises important treatment issues, as Drs. Jon E. Grant, Matt G. Kushner, and Suck Won Kim explain. For example, treatment of either disorder could be complicated or even compromised by the presence of the other untreated condition. The frequency of this comorbidity highlights the importance of screening for pathological gambling among patients being treated for alcohol use disorders and vice versa. (pp. 143–150)

## EATING DISORDERS AND ALCOHOL USE DISORDERS

Research to date––including studies of brain chemistry and family and genetic studies––has not established a firm link between eating disorders and alcohol use disorders. Nevertheless, as noted by Drs. Carlos M. Grilo, Rajita Sinha, and Stephanie S. O’Malley, the reality that eating disorders and alcohol use disorders frequently co-occur has important implications for assessment, treatment, and future research. The authors examine the extent and nature of co-occurring alcohol use disorders and eating disorders and the possible connections between them. They also review research that may help clinicians identify the presence of comorbid problems and determine the best treatment approach, including whether to address one problem first or to address both concurrently. (pp. 151–160)

